# Etoxazole Exposure Triggers Sublethal Metabolic Responses in Earthworms (*Eisenia fetida*): An NMR-Based Metabolomics Study

**DOI:** 10.3390/toxics13110923

**Published:** 2025-10-28

**Authors:** Chaoxuan Liao, Qinghai Zhang, Zelan Wang, Zuyong Chen, Yu He, Ji He, Dali Sun

**Affiliations:** 1 Guizhou Academy of Testing and Analysis, Guiyang 550014, China; liaochaoxuan@gzata.cn (C.L.); heyu@gzata.cn (Y.H.); 2 School of Public Health, Guizhou Medical University, Guiyang 561113, Chinawangzelan@gmc.edu.cn (Z.W.); 3 College of Agriculture, Guizhou University, Guiyang 550025, China

**Keywords:** etoxazole, metabolomics, nuclear magnetic resonance, earthworm, biomarker

## Abstract

As a highly effective acaricide, etoxazole is widely used in agricultural production, but its toxicological effects on soil organisms remain unclear. Based on nuclear magnetic resonance (NMR) metabolomics technology, in this study, we systematically investigated the sublethal responses of earthworms (*Eisenia fetida*) to etoxazole. The results showed that etoxazole exposure significantly altered the endogenous metabolic profiles in earthworms, with 19 and 20 metabolites significantly changed after 2 and 14 d of exposure, respectively. Trimethylamine N-oxide exhibited specific changes, indicating that it may be a potential biomarker for exposure to etoxazole. KEGG pathway analysis revealed that five metabolic pathways were notably affected after 2 and 14 days of etoxazole exposure. These pathways were primarily associated with energy conversion, protein and amino acid synthesis and metabolism, carbohydrate metabolism, and nucleic acid and DNA synthesis. Overall, etoxazole exposure notably altered the endogenous metabolic profiles of earthworms. This study was of great significance for comprehensively understanding the potential hazards that etoxazole poses to soil ecosystems and provides important information for environmental monitoring and ecological risk assessment.

## 1. Introduction

**Figure 1:**
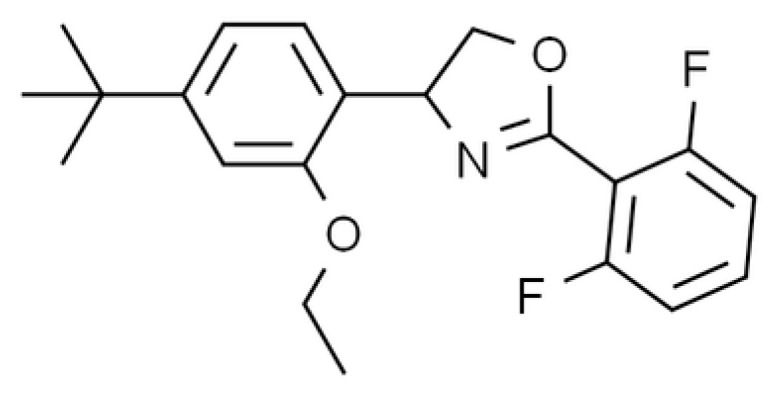
Chemical structure of etoxazole.

Etoxazole, 2-(2,6-difluorophenyl)-4-[4-(1,1-dimethylethyl)-2-ethoxy phenyl]-4, 5-dihydrooxazole, is a type of 2,4-diphenyl-1,3-oxazoline pesticide, and its chemical structure is as Figure 1:

It exhibits high efficacy against the eggs, larvae, and nymphs of various mites, such as *Panonychus citri*, *Phyllocoptruta oleivora*, and *Polyphagotarsonemus latus* [1]. Meanwhile, it can also be used as a growth regulator for insects or mites by inhibiting their growth cycles [2,3]. Since 2012, etoxazole has been registered in China and widely used in citrus, cotton, apple, and vegetables to control various kinds of mites [4]. According to literature reports, the half-life of etoxazole was approximately 24.2 days in soil [5], and the 45 days exposure experiment showed that etoxazole gradually decreased in soil samples by 28% of the initial amounts [6]. To date, studies of etoxazole have mainly been focused on its residue in agricultural products and toxicity effects. To determine etoxazole residues in apples, strawberries, green beans, and red pepper, methods using high-performance liquid chromatography (HPLC), gas chromatography (GC), and liquid chromatography–mass spectrometry (LC-MS/MS) have been established [7,8,9], which can be used to study its dissipation behavior in environment and evaluate the potential risk to humans. Meanwhile, the toxicity of etoxazole to cells, fish, and rats had also been reported. Etoxazole can reduce cell activities and alter lactate dehydrogenase (LDH) release levels, reactive oxygen species (ROS) generation, superoxide dismutase (SOD) and catalase (CAT) activities, and malonaldehyde (MDA) levels in cells and rats [10,11,12]. The effects of etoxazole exposure on embryogenesis and cardiovascular development in zebrafish have also been reported [13]. However, these studies were mainly focused on one or several indictors which cannot accurately and comprehensively evaluate the toxicity effect of etoxazole. Meanwhile, its mechanism of action on non-targeted organisms is still unclear.

Metabolomics technology can reflect the changes in endogenous small molecules in organisms due to physiological changes or exogenous stress including exposure to xenobiotic pollutants. These significantly changed molecules can be used to screen out potential biomarkers and study the modes of toxic action. Metabolomics has been widely used in disease diagnosis, gene function evaluation, toxicity evaluation, and so on [14]. In terms of toxicity research, potential biomarkers have been determined by comparison to different metabolites between treated groups and control groups in order to identify the metabolic pathways that may be affected in organism and evaluate the toxic mechanism at work [15].

Due to the species diversity of the endogenous substances and the complexity of the sample matrix, instruments with high precision and accuracy are required. With the development of modern science and technology, high-sensitivity and high-resolution instruments have gradually been applied to metabolomic study, including gas chromatography–mass spectrometry (GC-MS), LC-MS/MS, capillary chromatography (CE-MS), and nuclear magnetic resonance (NMR). Compared to the other three instruments, NMR has the advantages of high repeatability, no requirement for standards, and no loss of samples, which means that it can realize nondestructive detection and be used to separate compounds in complex sample matrices in a short time [16]. Since the 1980s, NMR has been widely used in toxicology research to monitor biomarkers related to toxicity in biological or cell samples in order to identify toxicity targets and modes of action in various organisms [17].

Metabolomics plays an important role in the safety evaluation of pesticide toxicity. It can comprehensively analyze the effects of pollutants on organisms at different concentrations or different exposure periods [18]. The toxicity of benalaxyl to mice was evaluated by a 30-day gavage exposure based on ^1^H NMR metabolomics. The results showed that the levels of histidine, asparagine, and lysine were significantly altered, and the energy metabolism, lipid metabolism, and urea cycle pathways were notably disturbed after benalaxyl treatment [19]. Sublethal levels of organochlorine pesticides DDT and chlordane were reported on the metabolic profiles of earthworms. Compared with the control group, the contents of maltose, leucine, and alanine in earthworms were significantly changed, and it was further confirmed by GC-MS that alanine may be the biomarker of DDT and chlordane exposure in earthworms [20]. The toxicity mechanism of endosulfan and its metabolite endosulfan sulfate in earthworms at concentrations of 0.1, 1.0, and 10 mg/kg dw was determined by one- and two-dimensional NMR-based metabolomics. The metabolites of glutamate/GABA glutamine in earthworms were significantly affected, indicating their neurotoxicity and apoptosis [21]. Thus, NMR-based metabolomics could be an appropriate tool to analyze the toxic effects of pesticides on organisms at the molecular level.

Until now, the metabolic responses of organisms to etoxazole have been unclear. Earthworms are key indicator organisms for soil health. *Eisenia fetida*, in particular, is widely used in toxicological testing owing to its global distribution, ease of cultivation, high fecundity, and sensitivity to toxicants. Therefore, in this study, the toxic effects of etoxazole on earthworms under different concentrations and different exposure periods were systematically assessed using ^1^H NMR-based metabolomics. The metabolites were identified based on NMR spectra through an online database. The significantly changed endogenous metabolites and altered pathways in earthworms were then screened out to analyze the mode of action and the toxic mechanism after etoxazole exposure. The results of this study can provide important and comprehensive insights into etoxazole toxicology in order to develop prevention strategies at an early stage.

## 2. Materials and Methods

### 2.1. Earthworm Exposure

Artificial soil was prepared according to the OECD earthworm acute toxicity test protocol [22]; it contained 70% sand, 20% clay, and 10% moss. According to the International union of pure and applied chemistry (IUPAC), the acute toxicity LC_50_ (14-day) of etoxazole to earthworms is >1000 mg/kg dry weight in soil, and the NOEC for 56-day reproduction toxicity is 5 mg/kg dry weight in soil [23]. Based on these endpoints, test concentrations of 10 (2 times of the NOEC), 50, and 100 mg/kg dry weight in soil were applied in this study. The soil was then dried under natural conditions and 100 g (dry weight) was weighed into a 1 L beaker. The formulation of 40% etoxazole suspension concentrates (SCs) was dissolved in water, then added into beakers, followed by the addition of another 400 g soil, with final etoxazole concentrations of 10, 50, and 100 mg/kg dw soil. The control group was prepared by adding the same amount of water. Three replicates were carried out for each treatment. The soil in all beakers was adjusted to a moisture of 36% and was replenished every two days.

Earthworms (*Eisenia fetida*), aged three months, were purchased from Organic Green Living Pte. Ltd. (Singapore), and were conditioned in the laboratory for one week before being used for the experiment. Ten earthworms with an average weight of 0.50 ± 0.08 g were placed into each one of the beakers mentioned above. Six earthworms were taken out at 2 and 14 days post-exposure for 24 h depuration.

### 2.2. Sample Extraction

Earthworms were weighed, frozen by liquid nitrogen, and then ground with a grinder. Samples of 0.1 g were weighed into 2 mL tubes, followed by the addition of 0.4 mL methanol, 0.4 mL chloroform, and 0.2 mL pure water under an ice bath. They were mixed in a vortex and then extracted for 10 min using ultrasound-assisted extraction. The extract was centrifuged at 4 °C for 10 min with a centrifugal force relative centrifugal force (RCF) of 2328 g. The supernatant was transferred into another 2 mL tube and lyophilized overnight. The lyophilized samples were reconstituted in phosphate-buffer solution (0.1 M, pH 7.4, with 10% D_2_O) with 0.5 mM chemical shift indicator (Disuccinimidyl suberate, DSS) added first for NMR analysis.

### 2.3. NMR Analysis

The spectra of all samples were acquired under a 600 MHz NMR spectrometer (Varian, Palo Alto, CA, USA). Each spectrum consisted of 128 scans and 16 384 data points in the frequency domain, and was obtained using water suppression (with a relaxation delay of 2.00 s), followed by a Carr–Purcell–Meiboom–Gill (CPMG) pulse sequence. The spectra were automatically Fourier-transformed using an exponential window with a line broadening value of 0.5 Hz, phased, and baseline-corrected using Chenomx NMR suite 7.6 (Chenomx Inc., Edmonton, AB, Canada). ^1^H NMR chemical shifts in the spectra were referenced to the DSS methyl peak at d 0.00. Under this condition, the signal region of H_2_O/D_2_O was at the 4.7–4.9 ppm bandwidth and was excluded from the spectral acquisition process.

### 2.4. Data Process

The NMR data were imported into SIMCA-P 12.0 software (MKS Instruments Inc., Umetric, Switzerland) for multivariate data analysis. Principal component analysis (PCA) and orthogonal partial least squares discriminant analysis (OPLS-DA) were performed based on the obtained data. For the OPLS-DA model, the values of X and Y were reported to indicate the fitting of training data. The NMR spectra were baseline-corrected and shimmed, and H_2_O/D_2_O signals (4.6–4.9 ppm) with width bins of 0.05 ppm were excluded. PCA was performed at the 95% confidence level. Multiple t-tests were constructed between the control and treated groups. SPSS software was used for multivariate analysis to identify the metabolites that exhibited significant differences from the control group. Variable importance in projection (VIP) was used to screen valuable biomarkers that may be closely related to toxicity from various metabolites. VIP > 1 indicated that the corresponding metabolites made a significant contribution to the clustering model. The screened-out metabolites were then analyzed by one-way analysis of variance (ANOVA) and Tukey’s test, which were carried out using IBM SPSS Statistics 21.0 (New York, NY, USA). Metabolites with VIP > 1 and *p* < 0.05 were considered potential biomarkers and were further imported to the KEGG database (https://www.kegg.jp/) and MetaboAnalyst 4.0 (http://www.metaboanalyst.ca/) to evaluate the disturbed pathways.

## 3. Results

### 3.1. NMR Method Development

**Figure 2:**
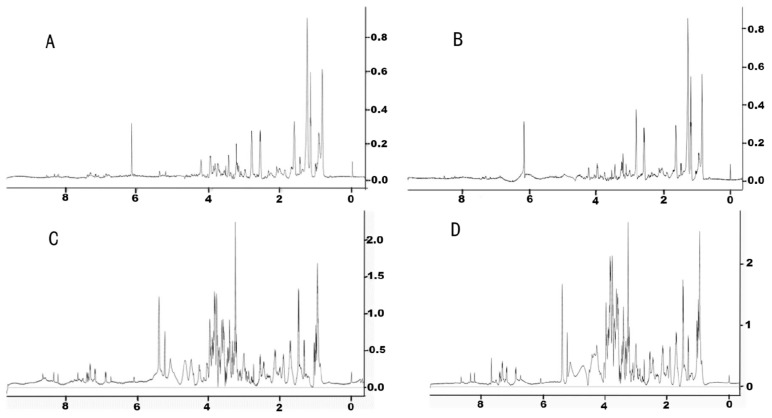
Optimization of endogenous metabolites extraction method for NMR analysis. (**A**) methanol/water (*v*/*v* = 1:1), (**B**) acetonitrile/water (*v*/*v* = 1:1), (**C**) methanol/dichloromethane /water (*v*/*v*/*v* = 1:1:1), (**D**) methanol/dichloromethane/water (*v*/*v*/*v* = 1:1:0.5).

In this experiment, four different solvent ratios were applied to extract the endogenous metabolites of earthworms; they were methanol/water = 1:1 (*v*:*v*), acetonitrile/water = 1:1 (*v*:*v*), methanol/dichloromethane/water = 1:1:1 (*v*:*v*:*v*), and methanol/dichloromethane/water = 2:2:1 (*v*:*v*:*v*). The NMR spectrum of the methanol/water mixture extract is shown in Figure 2A. It can be seen from the spectrum that few chemical shift peaks are detected. When we replaced methanol with acetonitrile, the obtained NMR spectrum was similar to that extracted by the methanol/water mixture, with a few chromatographic peaks detected (Figure 2B). The extract solvent was then changed to the polar solvent dichloromethane, which was the mixture of methanol/dichloromethanol/water with a volume ratio of 1:1:1. The numbers of the shift peaks under this condition were clearly more than those extracted without dichloromethane (Figure 2C). On this basis, the volume ratio of the methanol/dichloromethane/water mixture was then optimized to 1:1:0.5 by increasing the proportion of the organic solvent. As shown in Figure 2D, the NMR spectrum obtained was not significantly different from that in Figure 2C, while the relative intensity of these metabolites increased. Therefore, the extraction solvent of the methanol/dichloromethane/water mixture with a ratio of 1:1:0.5 by volume was used for the extraction of earthworm metabolites. Under this condition, a total of 53 endogenous metabolites were monitored (Appendix A).

### 3.2. Multivariate Analysis of the Metabolic Response of Earthworms to Etoxazole

**Figure 3:**
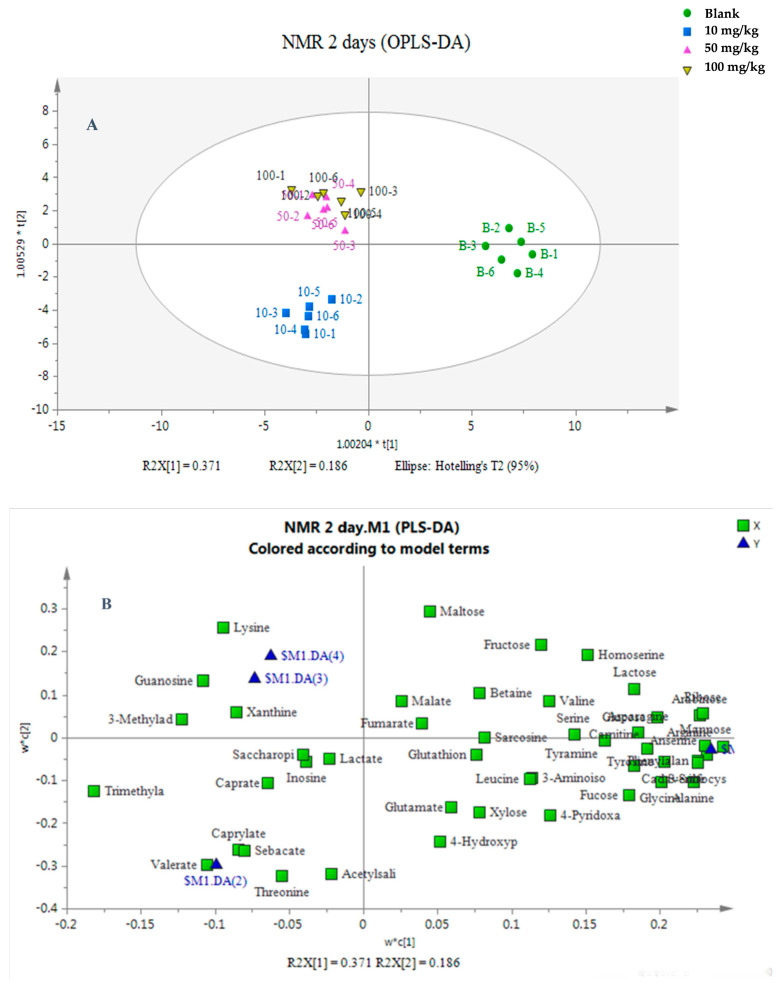

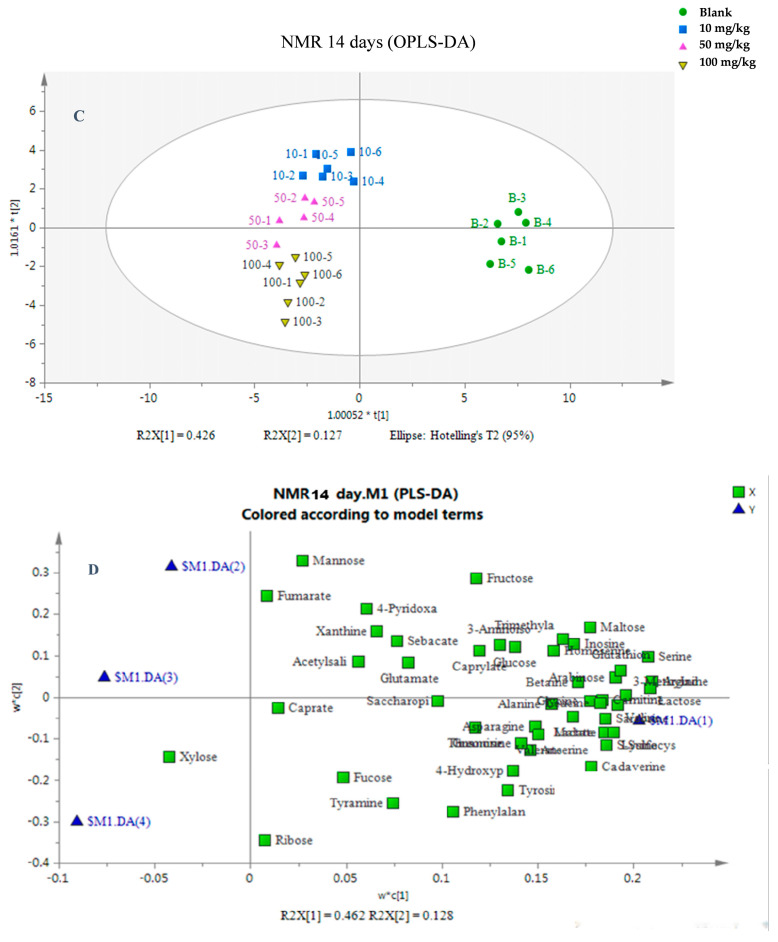
OPLS-DA (**A**,**C**) score plots and loading plots (**B**,**D**) of earthworm metabolites after etoxazole exposure at 0, 10, 50 and 100 mg/kg dw soil at 2 d (**A**,**B**) and 14 d (**C**,**D**). In the score plots (**A**,**C**), data points are presented as mean ± SD (n = 3). In the loading plots (**B**,**D**), each circle represents a metabolite. The size of the circle corresponds to the confidence level, with a larger size indicating a higher contribution and reliability of the metabolite to the model. The numerical prefixes on the axis labels (e.g., “1.00204” for t[1]) are scaling factors automatically applied by the software to optimize the graphical display of the score plot.

The identified metabolites were then imported into SIMCA-P software for data analysis. For OPLS-DA analysis, the values of R_2_X[1] were 0.371 and 0.426, and those R_2_X[2] were 0.186 and 0.127 for 2- and 14-day exposures, indicating that the first and second principal components can explain 55.7% and 55.3% of the variables, respectively. Three clusters were observed for the 2-day exposure, namely, a control group cluster, a low-concentration (10 mg/kg dw soil) cluster, and a medium- (50 mg/kg dw soil) and high-concentration (100 mg/kg dw soil) cluster, indicating no significant differences between the medium- and high-concentration groups (Figure 3A). On the other hand, for the 14-day exposure, four clusters were observed, indicating that the control group and the three treatment groups were completely separated (Figure 3C). The values of R_2_Y (0.856 and 0.978) and Q2 (0.864 and 0.720 for 2- and 14-day exposures, respectively) were used to explain the degree and indicate the predictive ability of the analysis method for the variables. Our results showed that the established method has good interpretation and prediction ability for original data. A loading plot was applied in all variables to evaluate the contributions of each variable according to its distance from the origin. The further away the variable was, the greater its contributions. After a 2-day exposure, the metabolites were distributed around the origin lines of the X and Y axes, while for the 14-day exposure, the metabolites were mainly located at the right of the X axis (Figure 3B,D). In this study, VIP values greater than 1 were filtered out. These metabolites were then analyzed by a t-test at a significance level of 95%. Under these conditions, 19 and 20 metabolites with significant differences were obtained for 2- and 14-day exposures.

The metabolomics study based on ^1^H NMR reflected the relationships between exposure period, exposure concentration, and the reactions of earthworms after being exposed to etoxazole. After 2 and 14 days of exposure, significant differences were observed in metabolic profiles between control group and treated groups (*p* < 0.05). OPLS-DA and loading plots were used to isolate metabolites with significant difference among different treatment groups. At 2 d, the results of OPLS-DA showed that three major clusters could be observed. The control group was located on the right side of the abscissa, while the three treated groups were on the left side of the abscissa, indicating that metabolites in earthworms were changed after treatment with etoxazole. The low-dose group (10 mg/kg dw soil) was completely separated from the other two groups and was located below the Y axis, while no significant difference was observed between the medium (50 mg/kg dw soil)- and high (100 mg/kg dw soil)-dose groups. A possible reason might be that the period of exposure to etoxazole was too short, and the difference between medium and high concentrations was not observed. At 14 d of exposure, completely separate clusters were observed between the control and treated groups, and the metabolic profiles in earthworms also showed a concentration-dependent cluster phenomenon. Compared with the 2 d treatment, the difference in metabolites in vivo caused by medium and high doses was more obvious after 14 d exposure.

### 3.3. Changes in Endogenous Metabolites in Earthworm After Etoxazole Treatment

**Figure 4:**
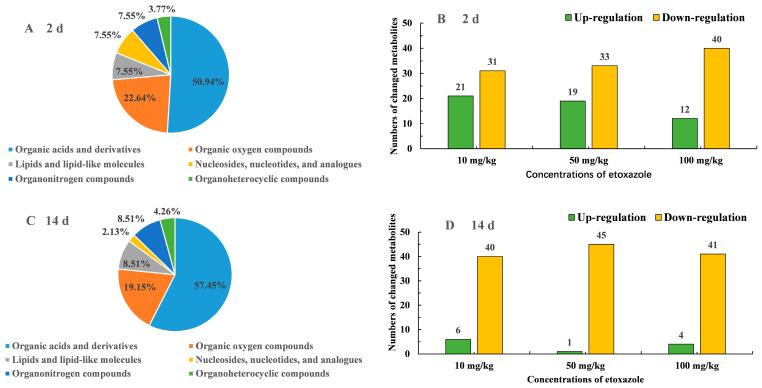
Proportions of the altered metabolites categories in earthworms after exposure to etoxazole for 2 d (**A**) and 14 d (**C**) and the numbers of up- and down-regulated metabolites compared to blank group at the etoxazole concentrations of 10, 50 and 100 mg/kg dw soil for 2 d (**B**) and 14 d (**D**).

After 2 and 14 days of exposure, 53 and 47 endogenous metabolites were detected, among which 19 and 20 metabolites were significantly changed compared to the control group under the conditions of VIP > 1 and *p* < 0.05. More than half of these metabolites, 50.94% and 57.45%, belonged to organic acids and derivatives, followed by organic oxygen compounds at 22.64% and 19.15%, and lipids and lipid-like molecules at 7.55% and 8.51% at 2 d and 14 d, respectively (Figure 4A,C). At 2 d of exposure, the number of down-regulated metabolites was higher than that of up-regulated metabolites compared to control group, with high dosage inducing more down-regulated metabolites (Figure 4B). At 14 d, most of the metabolites were down-regulated compared to control group (Figure 4D). These results indicated that higher concentration and a longer exposure period can decrease the content of metabolites.

**Figure 5:**
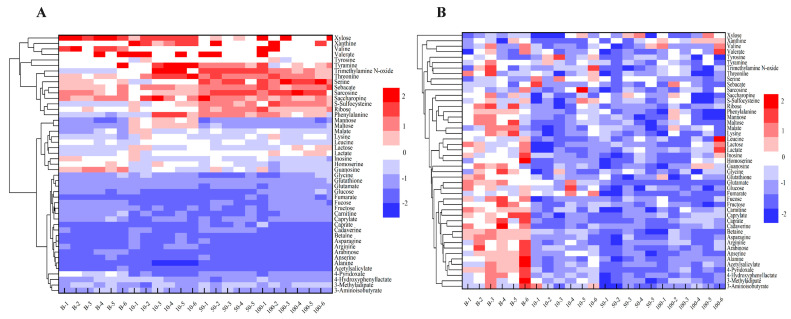
Heatmap generated by hierarchical clustering of the potential discriminating metabolites obtained from control, 10, 50, and 100 mg/kg dw soil etoxazole treatment for 2 d (**A**) and 14 d (**B**). The blue color gradient (from dark to light) indicated the metabolites with decreased content, whereas the red gradient (from light to dark) represented the metabolites with increased concentrations.

The heatmap analysis of the set of 47 shared metabolites (detected at both day 2 and day 14) showed that there was no significant difference among the control group, 10, 50, and 100 mg/kg dw soil etoxazole exposure at 2 d. The down-regulated metabolites were more than the up-regulated ones (Figure 5A). However, the metabolic profiles at 14 days of exposure remarkably decreased compared to control group (Figure 5B). These results showed that with the increase in exposure period, the differentiation among different groups increased, indicating that etoxazole can significantly affect metabolic profiles in earthworms.

**Figure 6:**
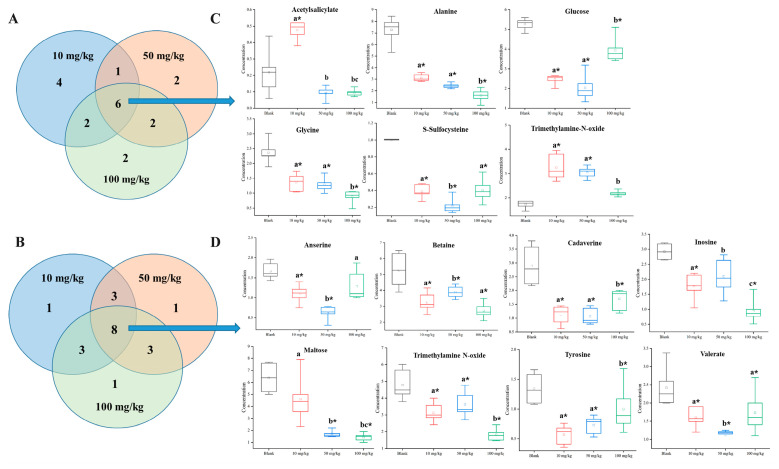
Venn diagrams for 10, 50, and 100 mg/kg dw soil extoxazole exposure, showing the numbers of shared and significantly changed metabolites in earthworms at 2 d (**A**) and 14 d (**B**). The squares define the sets for each concentration, and their overlaps illustrate the common metabolites across different treatments. Concentrations of commonly altered metabolites at 3 etoxazole concentrations (10, 50, and 100 mg/kg dw soil) for 2 d (**C**) and 14 d (**D**). Data are expressed as mean ± SD. * Indicates that there was a significant difference between treated group and control group (*p* < 0.05, *t*-test). Different letters indicated significant differences among 3 treated groups (*p* < 0.05, *t*-test).

Venn diagrams showed that a total of 19 significantly affected metabolites were filtered out under the conditions of VIP > 1 and *p* < 0.05 at 2 d (Figure 6A). Among these metabolites, 7 were organic acids and their derivatives (sarconsine, alanine, glycine, S-sulfocysteine, threonine, lysine, and malate), 4 were organic oxygen compounds (maltose, xylose, glucose, and fructose), 4 were lipids and lipid-like molecules (caprylate, sebacate, valerate, and caprate), 1 was a benzenoid (acetylsalicylate), 1 was an organoheterocyclic compound (xanthine), 1 belonged to the category nucleosides, nucleotides, and analogs (guanosine), and 1 was an organonitrogen compound (trimethylamine N-oxide). Caprate was a common metabolite in 10 and 50 mg/kg dw soil exposure, lysine and malate were the 2 metabolites that were shared by 50 and 100 mg/kg dw soil exposure, and fructose and threonine were shared by 10 and 100 mg/kg dw soil exposure. Six metabolites, namely, acetylsalicylate, alanine, glucose, glycine, S-sulfocysteine, and trimethylamine N-oxide, were commonly altered after 10, 50, and 100 mg/kg dw soil exposure (Figure 6C).

20 significantly altered metabolites were screened out at 14 d exposure (Figure 6B). Among them, 6 belonged to the category organic acids and their derivatives (tyrosine, phenylalanine, saccharopine, threonine, anserine, and 3-aminoisobutyrate), 6 were organic oxygen compounds (ribose, fucose, maltose, mannose, fructose, and glucose), 3 were organonitrogen compounds (cadaverine, betaine, and trimethylamine N-oxide), 1 belonged to the category lipids and lipid-like molecules (valerate), 1 was an organoheterocyclic compound (4-pyridoxate), 1 belonged to the category nucleosides, nucleotides, and analogs (inosine), 1 was a benzenoid (tyramine), and one belonged to the category phenylpropanoids and polyketides (4-hydroxyphenyllactate). Three metabolites, namely, 4-hydroxyphenyllactate, phenylalanine, and tyramine, were shared by 10 and 50 mg/kg dw soil exposure. Mannose, saccharopine, and threonine were the common metabolites in 50 and 100 mg/kg dw soil exposure. 3-aminoisobutyrate, fructose, and glucose were shared by 10 and 100 mg/kg dw soil exposure. Anserine, betaine, cadaverine, inosine, maltose, trimethylamine N-oxide, tyrosine, and valerate were the metabolites shared by 10, 50, and 100 mg/kg dw soil exposure (Figure 6D). The contents of all these metabolites were significantly decreased compared to the blank group.

### 3.4. Possible Metabolic Pathways in Earthworms Affected by Etoxazole

**Figure 7:**
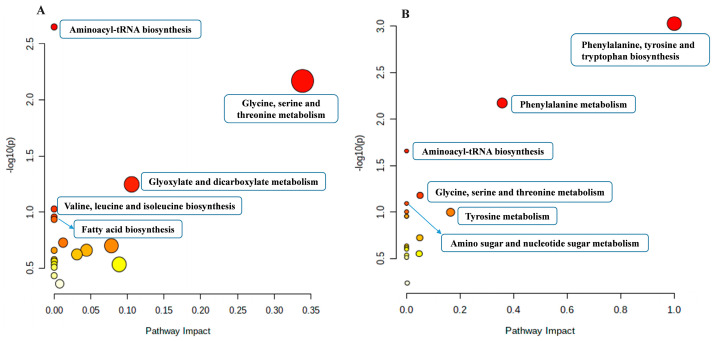
Overview of enriched metabolic pathways of earthworms after etoxazole exposure for 2 d (**A**) and 14 d (**B**). The color of the bubbles represents the −log10 (*p*-value), with a gradient from yellow to red signifying the increasing statistical significance. Their size corresponds to the pathway impact value, with larger bubbles the more serious the pathway perturbed.

The metabolic pathways of significantly altered metabolites were confirmed by using the KEGG database and MetaboAnalyst 4.0 software. Based on a comprehensive consideration of both the statistical significance (*p*-values) of pathway enrichment and the pathway impact values. The most affected metabolic pathways after 2 d of exposure were screened out which were aminoacyl-tRNA biosynthesis; glycine, serine, and threonine metabolism; glyoxylate and dicarboxylate metabolism; valine, leucine, and isoleucine biosynthesis; and fatty acid biosynthesis (Figure 7A). For 14 d of exposure, the top 5 affected metabolic pathways were phenylalanine, tyrosine, and tryptophan biosynthesis; phenylalanine metabolism; aminoacyl-tRNA biosynthesis; glycine, serine, and threonine metabolism; and amino sugar and nucleotide sugar metabolism (Figure 7B). Aminoacyl-tRNA biosynthesis and glycine, serine, and threonine metabolism were the two pathways shared by 2 d and 14 d exposure.

## 4. Discussion

As a highly effective acaricide, etoxazole has garnered significant attention due to its toxicity toward non-target organisms. To further investigate its toxic effects on earthworms, NMR technology was used to optimize metabolite extraction methods, examine the metabolic disruption mechanisms induced by etoxazole, and identify potential biomarkers. The methanol/water system is a widely used method for extracting metabolites in living organisms and has been extensively applied in the extraction of compounds from various animals such as rats and zebrafish [24,25]. In this study, the addition of dichloromethane—a nonpolar solvent—to the conventional methanol/water system significantly increased the number of detectable chemical shift peaks, leading to the identification of 53 endogenous metabolites. The number of metabolites detected by NMR-based metabolomics in this work was 26% more than that reported by Bao et al., who detected 42 metabolites using the methanol/water system [26]. This enhancement is likely due to the incorporation of dichloromethane, which improved the extraction rate of nonpolar compounds. The results of PCA and OPLS-DA showed that etoxazole significantly altered the endogenous metabolic profile of earthworms under different concentrations and exposure periods. The affected metabolites included organic acids and their derivatives, organo-oxygen compounds, lipids and lipid-like molecules, and other compounds such as cadaverine.

### 4.1. Organic Acids and Their Derivatives

After 2 d of etoxazole exposure, 7 organic acids and their derivatives, namely, S-sulfocysteine, alanine, glycine, threonine, lysine, sarcosine, and malate, were altered, and 6 of them were categorized as amino acids and their derivatives. Among them, S-sulfocysteine, alanine, and glycine were significantly decreased (*p* < 0.001) after 10, 50, and 100 mg/kg dw soil etoxazole exposure. S-sulfocysteine is the conjunction of S-sulfo and L-cysteine which can increase cell proliferation and has an anti-oxidative potential through increasing the levels of superoxide dismutase enzyme and total intracellular glutathione [27]. Moreover, the excess accumulation of S-sulfocysteine can be excitotoxic and is involved in many neuropathological disorders like sulfite oxidase deficiency that may lead to neurological dysfunction [28]. In this study, the content of S-sulfocysteine decreased indicating that etoxazole may inhibit the growth of earthworms and remarkably weaken S-sulfocysteine’s anti-oxidative capacity. Alanine plays a key role in glucose–alanine cycle with the intermediate products of pyruvate and α-ketoglutarate. Pyruvate can be used in gluconeogenesis to form glucose and transport it back to tissues and organs by circulatory system. α-Ketoglutarate is an important substance in tricarboxylic acid cycle (TAC), providing energy to organisms [29,30]. In this study, the content of alanine significantly decreased (*p* < 0.001), indicating the lack of energy metabolism. Glycine can be used as a cell-protective agent to prevent damage by harmful substances to the cells, tissues, and organs of organisms. For example, glycine has an antagonistic effect towards endotoxin, effectively preventing it from damaging the body [31,32,33]. In this study, the content of glycine decreased significantly (*p* < 0.001) with the increase in etoxazole dosage. The decrease in glycine content may lead to the loss of protective agents, which may induce the entrance of endotoxin or external toxins, resulting in damage to the body. Moreover, the decrease in glycine content may lead to obesity and an increase in blood lipids, leading to related metabolic diseases. However, the other two amino acids, threonine and lysine, were significantly increased compared to control group (*p* < 0.05). Threonine is an essential amino acid that participates in the construction of the immune system [34,35]. In this study, the relative content of threonine increased first and then decreased compared to control group after 2 days of exposure. The increased threonine level was mainly used to enhance immunity. Lysine is an important essential amino acid in vivo which is involved in protein biosynthesis and energy metabolism [36]. In this study, the relative content of lysine significantly increased with the increase in etoxazole content, which may be used to compensate for damage to the body after etoxazole exposure.

After 14 days of exposure, 6 organic acids and their derivatives, namely, tyrosine, phenylalanine, saccharopine, threonine, anserine, and 3-aminoisobutyrate, were altered. The content of all these metabolites decreased after exposure to etoxazole, indicating the disorder of organic acid metabolism and somatic function in earthworms. Tyrosine and phenylalanine significantly decreased after 10, 50, and 100 mg/kg dw soil etoxazole exposure. Phenylalanine can be converted to tyrosine, which is closely related to nerve conduction [37]. Therefore, phenylalanine plays an important role in maintaining a normal nervous system. In this study, the content of phenylalanine in earthworms decreased significantly after exposure to different concentrations of etoxazole (*p* < 0.05), which may lead to damage to the nervous system of earthworms. Anserine, also known as beta-alanyl-3-methyl-L-histidine, is also related to memory function. The content of anserine significantly decreased after etoxazole exposure, also illustrating the effects of etoxazole on the nervous system [38].

### 4.2. Organic Oxygen Compounds

Four organic oxygen compounds, namely, maltose, glucose, fructose, and xylose, were significantly altered after 2 d of exposure. Six organic oxygen compounds, namely, maltose, glucose, fructose, ribose, fucose, and mannose, were significantly decreased, except mannose and fructose, which increased at a concentration of 10 mg/kg dw soil. Maltose, glucose, and fructose were the three organic oxygen compounds that were shared by 2- and 14-day exposure. Most of these compounds first significantly decreased and then increased. Compared to control group, the content of glucose significantly decreased after etoxazole exposure. However, the degree of decrease slowed down after exposure to a high dose of etoxazole, which may be due to the conversion of other energy substances such as lipids into carbohydrates. The content of maltose decreased after treatment with a low concentration of etoxazole for 2 days, which may be due to the high glycogen consumption. However, the content of maltose increased with the increase in etoxazole content, indicating the distribution of carbohydrate metabolism, which led to the accumulation of maltose. However, after 14 days of treatment, maltose content decreased significantly with the increase in exposure concentration, indicating the large amount of maltose consumption in earthworms. The contents of fructose in the two exposure periods were both significantly decreased (*p* < 0.05), which may weaken the rate of glucometabolism and lead to a loss of bodyweight. However, the relative content of fructose after 14 days of exposure was greater than after 2 days of exposure, showing that more serious damage was observed after long-term exposure. The contents of the other 4 organic oxygen compounds xylose, ribose, fucose, and mannosewere related to the production of adenosine monophosphate and ATP and energy metabolism, were all decreased at 3 etoxazole concentration levels [39]. These phenomena can be explained by the lack of glycogen supply in earthworms, which may result in lack of energy, impairment of body function, and the slowdown of various metabolisms.

### 4.3. Lipids and Lipid-like Molecules

Four lipids and lipid-like molecules, namely, valerate, caprylate, sebacate, and caprate, were altered after 2 d of exposure. The contents of these metabolites significantly increased in low-dose group and then decreased to normal levels in high-dose group (*p* < 0.05). Valerate was the only lipid detected at both 14 d of exposure and 2 d of exposure. The content of valerate increased at 14 d compared to 2 d of exposure, indicating the lipid’s accumulation in earthworms. On the other hand, with the increase in etoxazole, the content of valerate significantly decreased (*p* < 0.05) at 14 d. Valerate, caprylate, sebacate, and caprate belong to the family of fatty acids, and their main function is to provide energy [40]. When cells undergo starvation, the fatty acids can be used and transferred to ATP to provide energy through the process of oxidation [41]. The significant increase in these four fatty acids at a low etoxazole concentration might be due to the earthworms’ need for energy replenishment at 2 d of exposure. On the other hand, the content of valerate significantly decreased at 14 d of exposure, indicating the lack of energy supply in earthworms.

### 4.4. Other Compounds

After 2 days of exposure, level changes were observed for cadaverine, inosine, 4-hydroxyphenyllactic acid (4-HPL), and betaine. Alterations were also detected in the disease-associated metabolite trimethylamine N-oxide. By 14 days of exposure, the levels of the above metabolites significantly increased compared to 2 days of exposure. This suggested that the transition from the cultivation system to the experimental environment induced stress in earthworms, which subsequently up-regulated these protective metabolites to facilitate self-protection or physiological adaptation.

Cadaverine is a biogenic amine generated from L-lysine via decarboxylation catalyzed by lysine decarboxylase (LDC) [42]. Elevated cadaverine levels may indicate enhanced microbial activity [43]. Cadaverine has also been proposed to function as a protective metabolite, whose synthesis capacity significantly declines in early-stage (Stage 0 and I) breast cancer [44]. Additionally, cadaverine is involved in the regulation of gene expression and translation, indirectly influencing stress responses. Its accumulation under oxidative stress represents a preemptive antioxidant strategy mediated by metabolic plasticity [45]. After 2 days of exposure, cadaverine content in the 10 and 50 mg/kg dw soil treatment groups significantly increased compared to the control group (*p* < 0.05). These results suggested that short-term, low-concentration exposure transiently enhanced the earthworms’ antioxidant and self-protective capacities, whereas prolonged or high-concentration exposure reduced oxidative stress tolerance, weakened self-defense ability, and increased susceptibility to disease or mortality.

4-Hydroxyphenyllactic acid (4-HPL) was consistently detected under all etoxazole treatments (10, 50, and 100 mg/kg dw soil) at both 2 and 14 days. This metabolite, present in environments such as the intestine, is a characteristic homeostatic end product of tyrosine metabolism and exhibits antifungal activity [46,47]. After 2 days of exposure, 4-HPL levels decreased significantly in 3 treatment groups, indicating that etoxazole disrupted tyrosine metabolism in earthworms, affected microbial community structure, and reduced antifungal capacity. The further reduction in 4-HPL after 14 days suggested progressive organismal damage, diminished tyrosine metabolic function, and increased vulnerability to fungal infection.

Inosine, which was notably decreased after both 2 and 14 days of exposure, is an endogenous purine nucleoside produced from adenosine via deamination. Studies indicated that inosine enhances mitochondrial respiratory lipolysis and oxygen consumption rate (OCR) [48], and exhibits context-dependent immunomodulatory functions—enhancing anti-tumor immunity in some settings while suppressing immune responses in others [47,49]. Etoxazole exposure at 10, 50, and 100 mg/kg dw soil resulted in significantly decreased inosine levels compared to the control group in two exposure periods, indicating the disrupted production or accumulation of inosine, which potentially compromised immune regulation and increased susceptibility to disease or death.

Betaine, a stable and non-toxic natural choline derivative found in seafoodwhich exhibits natural anti-inflammatory effects by limiting the secretion of inflammatory factors [50]. As an organic osmolyte, betaine can protect cells, proteins, and enzymes against environmental stressors such as desiccation, high salinity, and extreme temperatures. Betaine was detected in both control and treated groups at 2 and 14 days, and was significantly higher at 14 days than at 2 days, reflecting the earthworms’ adaptive production of betaine to cope with environmental stress. After 14 days of exposure, betaine content in all treatment groups significantly decreased compared to control group, indicating that etoxazole disrupted betaine synthesis or conversion. As an important anti-inflammatory compound and osmoprotectant. The decline in betaine may increase the risk of inflammation, and diminish stress tolerance thereby elevating overall physiological vulnerability.

Trimethylamine-N-oxide (TMAO) was commonly detected at 10, 50, and 100 mg/kg dw soil etoxazole exposure at both 2 and 14 d. TMAO is a potential biomarker for disease risk, closely linked to intestinal microbiota metabolism. Studies had been demonstrated that TMAO contributes to the pathogenesis of various diseases by disrupting host lipid homeostasis and promoting inflammation. Circulating TMAO levels are strongly linked to the development of metabolic syndromes and neurodegenerative disorders. Consequently, the concentration of TMAO is a significant predictor of disease progression and mortality, highlighting its potential in early warning systems [51]. The higher the concentration of TMAO in blood, the higher the disease or death risk. In this study, the content of TMAO in earthworms was significantly increased compared to control group after exposure for 2 days. With the increase in the concentration of etoxazole, the content of TMAO decreased. The significant increase in TMAO content indicated that the earthworms may produce the related disease traits, and the individual death risk is increased. After 14 days of exposure, TMAO content was reduced in all treatment groups, suggesting that the earthworms may have developed partial tolerance to etoxazole, correspondingly lowering the risks of disease or death.

### 4.5. Possible Metabolic Pathways

After exposure to etoxazole, metabolites with significant difference were subjected to pathway analysis using the KEGG pathway database (https://www.genome.jp/kegg/pathway.html, accessed on 15 August 2024). It was found that these carbohydrate compounds, amino acids, and biological small molecules not only participated in their own metabolism but were also involved in some other in vivo metabolism. These metabolites were mainly related to energy conversion, amino acid synthesis and metabolism, carbohydrate metabolism, nucleic acid DNA synthesis, and so on. Therefore, etoxazole mainly affected the normal physiological and biochemical activities of earthworms. After treatment with other pollutants, such as pesticides, heavy metals, and persistent environmental pollutants, the concentrations of the above metabolites also changed [52,53,54]. However, trimethylamine-N-oxide was not found after treatment with other pollutants, so it is a characteristic metabolite in earthworms caused by exposure to etoxazole.

## 5. Conclusions

In this study, NMR-based metabolomics was applied to systematically investigate the sublethal toxic effects and metabolic regulation mechanisms in earthworms (*Eisenia fetida*) under varying etoxazole concentrations and exposure durations. Our study showed that etoxazole exposure significantly disturbed the endogenous metabolic profiles of earthworms and impaired their physiological functions. Notably, TMAO exhibited specific alterations in all exposure groups, indicating its potential as a biomarker for etoxazole exposure and providing a molecular basis for pesticide environmental risk assessment. This study presented the first metabolic-level insight into the sublethal effects of etoxazole on earthworms, offering valuable data for evaluating its potential hazard to soil organisms and supporting pesticide safety evaluation. Future work could incorporate transcriptomic, proteomic, and other multi-omics approaches, along with field validation, to further elucidate the ecological risks of etoxazole.

## Data Availability

The original contributions presented in the study are included in the article, further inquiries can be directed to the corresponding author.

## Abbreviations

The following abbreviations are used in this manuscript:

NMR	Nuclear magnetic resonance
PCA	Principal component analysis
OPLS-DA	Orthogonal partial least squares-discriminant analysis
VIP	Variable importance in projection
TMAO	Trimethylamine N-oxide
HPLC	High-performance liquid Chromatography
GC-MS	Gas chromatography-mass spectrometry
LC-MS/MS	Liquid chromatography-tandem mass spectrometry
ROS	Reactive oxygen species
SOD	Superoxide dismutase
CAT	Catalase
MDA	Malonaldehyde
LDH	Lactate dehydrogenase
LC_50_	Lethal concentration 50
NOEC	No observed effect concentration
GABA	γ-aminobutyric acid
CPMG	Carr–Purcell–Meiboom–Gill
ATP	Adenosine triphosphate
4-HPL	4-hydroxyphenyllactic acid
OECD	Organisation for economic co-operation and development
SCs	Suspension concentrates
DSS	Disuccinimidyl suberate
ANOVA	Analysis of variance.

## Appendix A

**Table A1:** List of disturbed 47 endogenous metabolites.

Organic Acids and Derivatives	Organo-Oxygen Compounds (mainly Sugars)	Amino Acids, Peptides, and Analogs	Amines and Nitrogen-Containing Compounds	Lipids and Lipid-like Molecules	Nucleosides, Nucleotides, and Metabolites	Disaccharides
3-Aminoisobutyrate	Arabinose	Alanine	Betaine	Caprate	Guanosine	Lactose
3-Methyladipate	Fructose	Arginine	Cadaverine	Caprylate	Inosine	Maltose
4-Hydroxyphenyllactate	Fucose	Asparagine	Carnitine	Sebacate	Xanthine	None
4-Pyridoxate	Glucose	Glutamate	S-Sulfocysteine	None	None	None
Acetylsalicylate	Mannose	Glycine	Trimethylamine N-oxide	None	None	None
Fumarate	Ribose	Homoserine	Tyramine	None	None	None
Lactate	Xylose	Leucine	Saccharopine	None	None	None
Malate	None	Lysine	None	None	None	None
Valerate	None	Phenylalanine	None	None	None	None
None	None	Sarcosine	None	None	None	None
None	None	Serine	None	None	None	None
None	None	Threonine	None	None	None	None
None	None	Tyrosine	None	None	None	None
None	None	Valine	None	None	None	None
None	None	Glutathione	None	None	None	None
None	None	Anserine	None	None	None	None
